# Crystal structure and Hirshfeld-surface analysis of an etoxazole metabolite designated R13

**DOI:** 10.1107/S2056989024010600

**Published:** 2024-11-08

**Authors:** Thaluru M. Mohan Kumar, Besagarahally L. Bhaskar, Prabhakar Priyanka, Thayamma R. Divakara, Hemmige S. Yathirajan, Sean Parkin

**Affiliations:** a Department of Physical Sciences, Amrita School of Engineering, Amrita Vishwa Vidyapeetham, Bengaluru-560 035, India; b Department of Physical Sciences, Amrita School of Engineering, Amrita Vishwa Vidyapeetham, Bengaluru-560 035, India; c National Hill View Public School, Bengaluru-560 098, India; d Department of Chemistry, T. John Institute of Technology, Bengaluru-560 083, India; e Department of Studies in Chemistry University of Mysore, Manasagangotri Mysuru-570 006 India; f Department of Chemistry University of Kentucky,Lexington KY 40506-0055 USA; Institute of Chemistry, Chinese Academy of Sciences

**Keywords:** etoxazole metabolite R13, insecticide, acaricide, Hirshfeld surface analysis, crystal structure

## Abstract

The etoxazole metabolite **R13**, systematic name 4-(4-*tert*-butyl-2-eth­oxy­phen­yl)-2-(2,6-di­fluoro­phen­yl)oxazole (C_21_H_21_F_2_NO_2_), results from the oxidation of etoxazole, a chitin synthesis inhibitor belonging to the oxazoline class, widely used as an insecticide/acaricide since 1998. The structure of **R13** features a central oxazole ring with attached 2,6-di­fluoro­phenyl and 4-*t*-butyl-2-eth­oxy­phenyl moieties. The overall conformation gives dihedral angles between these rings and the oxazole of 24.91 (5)° (with di­fluoro­phen­yl) and 15.30 (6)° (with *t*-butyl-eth­oxy­phen­yl), indicating an overall deviation from planarity. Additionally, torsion angles of the eth­oxy and *t*-butyl groups define the orientation of these substituents relative to their benzene ring. In the crystal packing, no significant hydrogen bonds are present, but a Hirshfeld surface analysis highlights weak inter­molecular contacts leading to π–π-stacked dimers linked by weak C—H⋯N contacts. The packing analysis confirms that most inter­molecular inter­actions involve hydrogen atoms.

## Abstract 2

The crystal structure of a metabolite of the insecticide/acaricide etoxazole, designated R13 is presented along with a Hirshfeld surface analysis of inter­molecular inter­actions present in the crystal structure.

## 1. Chemical context

The etoxazole metabolite designated **R13**, systematic name 4-(4-*t*-butyl-2-eth­oxy­phen­yl)-2-(2,6-di­fluoro­phen­yl)oxazole (C_21_H_21_F_2_NO_2_), is derived from etoxazole (C_21_H_23_F_2_NO_2_), an organofluorine chitin synthesis inhibitor. Etoxazole is a member of the oxazoline class of insecticides, having been developed as a new-generation insecticide and acaricide (Li *et al.*, 2014 ▸). It has been used globally since 1998 (Park *et al.*, 2020 ▸). Etoxazole is readily absorbed by plants and translocates locally within leaves. The insecticidal mode of action of etoxazole is *via* the inhibition of chitin biosynthesis. A comprehensive review of the biological activities of oxazole derivatives was published by Kakkar & Narasimhan (2019 ▸), while Joshi *et al.* (2023 ▸) provided a detailed review of their chemistry. Recent research has also assessed the risks of oxidative stress and multiple toxicities induced by etoxazole (Macar *et al.*, 2022 ▸). The synthesis and activity of novel acaricidal/insecticidal 2,4-diphenyl-1,3-oxazolines were reported by Suzuki *et al.* (2002 ▸). It is well established that the key transformation of etoxazole in plants and animals involves oxidation of the oxazole ring, leading to the formation of the **R13** metabolite (APVMA, 2024 ▸).

We have recently reported the crystal structures of phenyl­pyrazole-based insecticides (Priyanka *et al.*, 2022 ▸; Vinaya *et al.*, 2023 ▸). The crystal structure of 2-(3-methyl-2-nitro­phen­yl)-4,5-di­hydro-1,3-oxazole, an inter­mediate in the synthesis of anthranilamide insecticides, was reported by Lei *et al.* (2009 ▸). Additionally, the crystal structure of ethyl 3-(4-chloro­phen­yl)-5-[(*E*)-2-(di­methyl­amino)­ethen­yl]-1,2-oxazole-4-carboxyl­ate was described by Efimov *et al.* (2015 ▸), and the structure of the insecticide fipronil was published by Park *et al.* (2017 ▸). Given the significance of etoxazole, we present in this paper the crystal structure of its metabolite, **R13**.
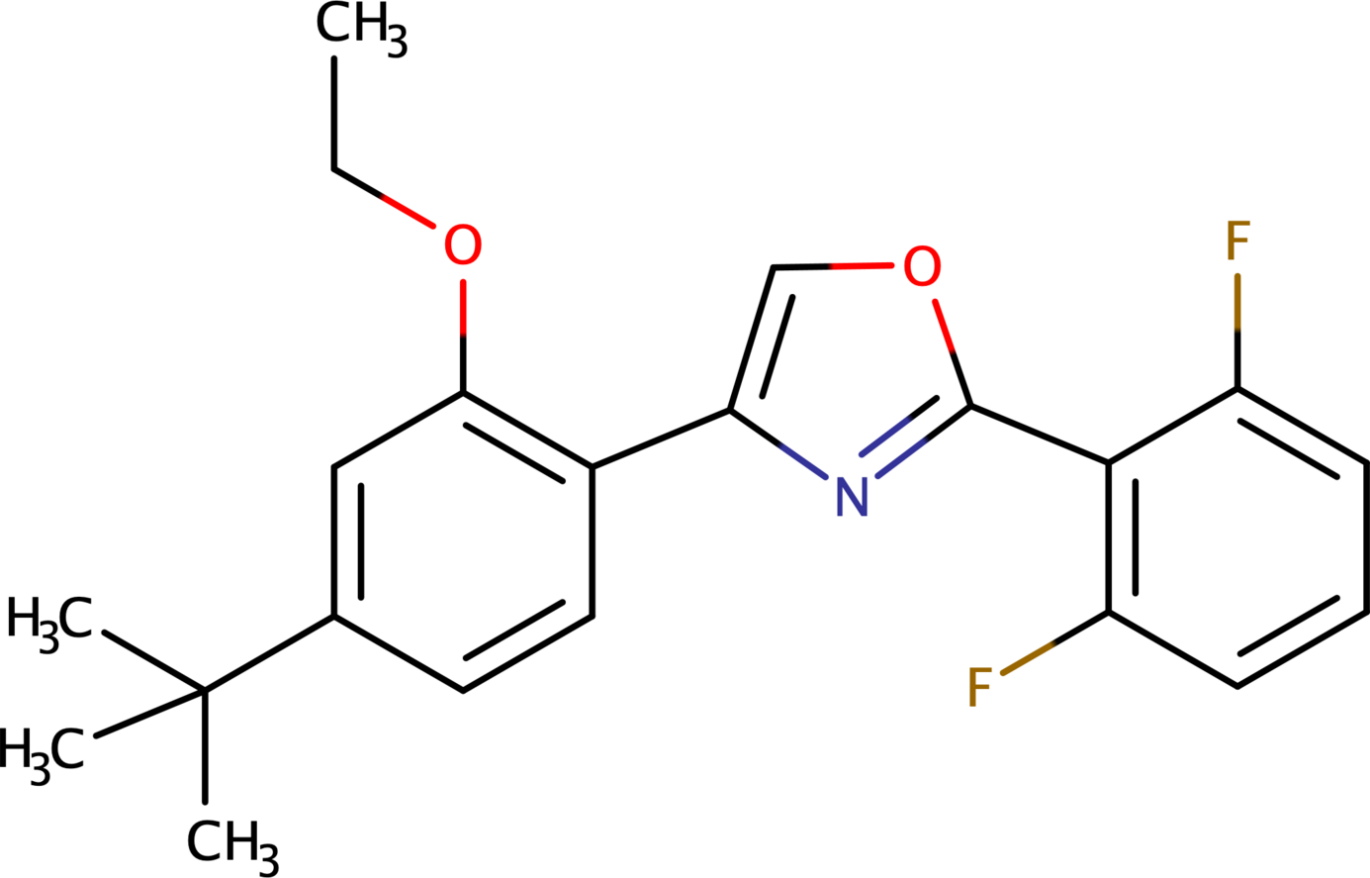


## 2. Structural commentary

**Figure 1:**
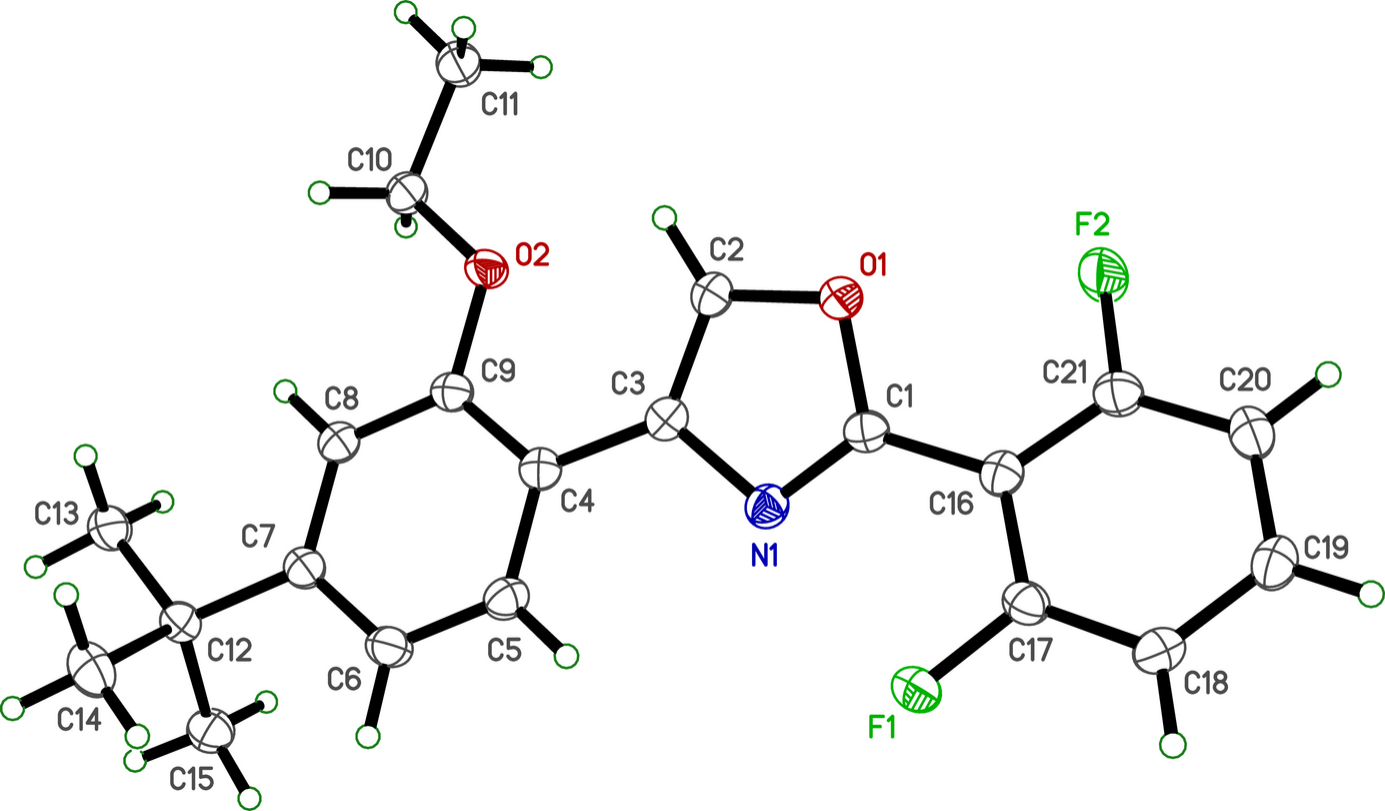
An ellipsoid plot of **R13** (50% probability). Hydrogen atoms are shown as circles of arbitrary radius.

The crystal structure of **R13** is monoclinic, space-group type *C*2/*c*. The mol­ecular structure (Fig. 1 ▸) consists of three substituted rings: a central oxazole ring flanked by a 2,6-di­fluoro­phenyl ring attached to the oxazole carbon between the nitro­gen and oxygen atoms, and a 4-*t*-butyl-2-eth­oxy­phenyl group attached to the oxazole carbon on the opposite side of the nitro­gen. There are no unusual bond lengths or angles within the mol­ecule.

The overall conformation is primarily defined by the dihedral angles between the oxazole and di­fluoro­phenyl rings [24.91 (5)°], and between the oxazole and *t*-butyl-2-eth­oxy­phenyl rings [15.30 (6)°]. The dihedral angle between the two benzene rings is 11.56 (6)°. These values indicate that the mol­ecule deviates from planarity, mostly due to the tilt of the oxazole ring relative to its attached substituents. An intra­molecular close contact between H2 and O2 (Table 1 ▸) is flagged as a ‘potential’ hydrogen bond by *SHELXL* (Sheldrick, 2015*b* ▸), but not by *Mercury* (Macrae *et al.*, 2020 ▸).

Further degrees of freedom in the structure are characterized by torsion angles, specifically the positioning of the eth­oxy group, defined by C4—C9—O2—C10 [176.53 (9)°] and C9—O2—C10—C11 [174.49 (9)°], and the relative orientation of the *t*-butyl group to its attached benzene ring, indicated by torsion C6—C7—C12—C13 [−177.08 (9)°].

## 3. Supra­molecular features

**Figure 2:**
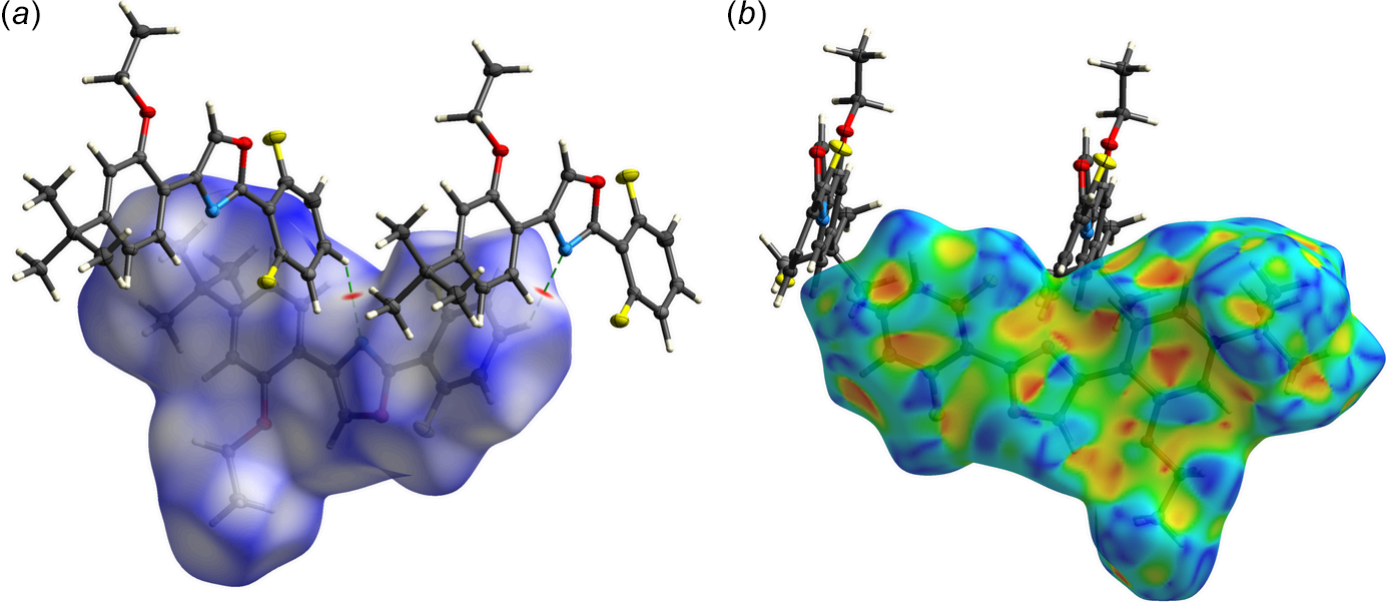
Two views of the Hirshfeld surface of **R13** showing: (*a*) the surface rendered as *d*_norm_, highlighting close contacts of the form C19^i^—H19^i^⋯N1 [symmetry code: (i) 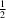 − *x*, 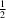 + *y*, 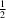 − *z*] as small red spots; (*b*) the surface rendered by ‘shape index’, which provides evidence of π–π stacking as opposing blue and red roughly triangular regions at each of the oxazole and benzene rings.

**Figure 3:**
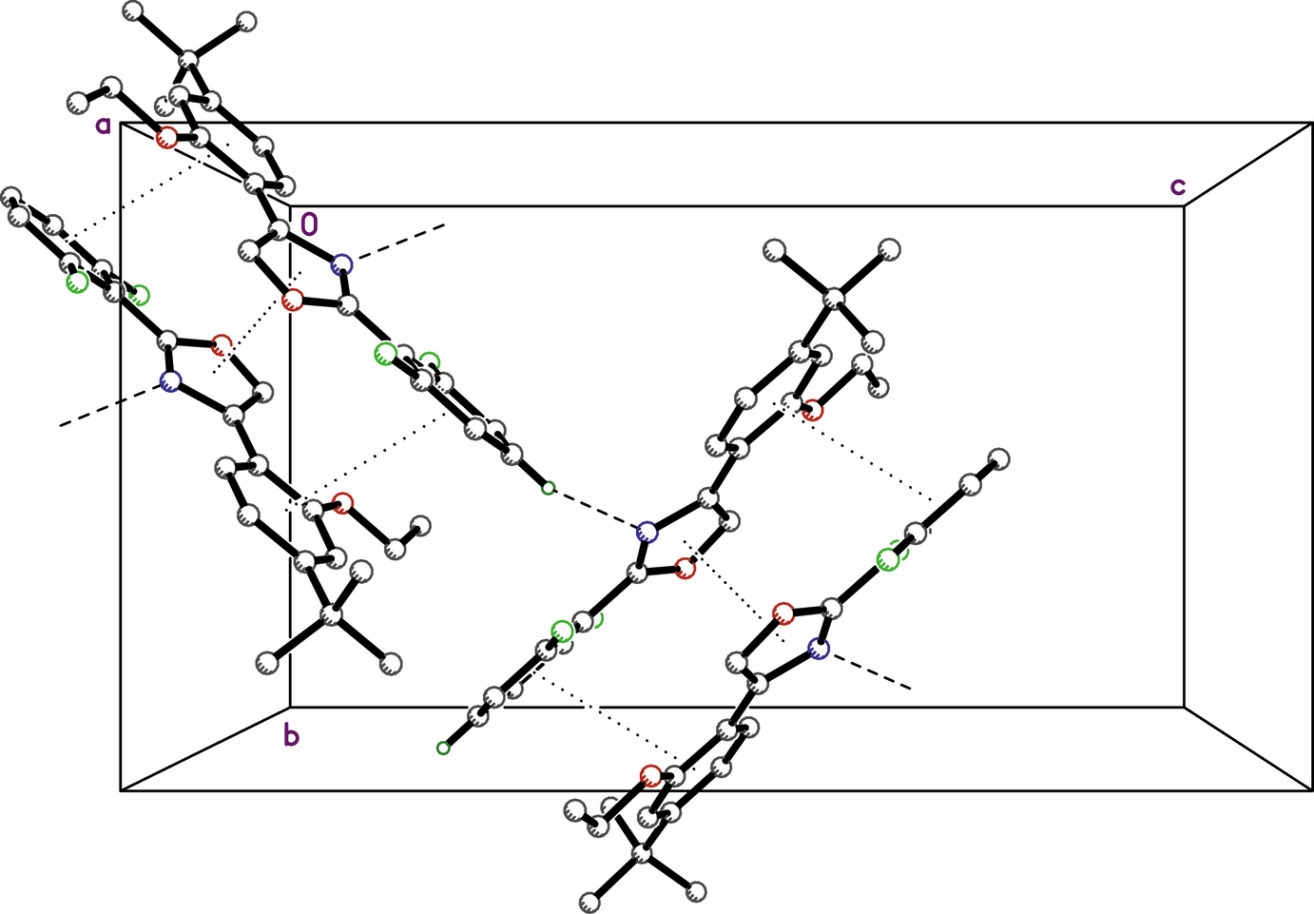
A partial packing plot of **R13** showing dimers resulting from the π–π stacking (indicated by dotted lines), and close C—H⋯N contacts between dimers (dashed lines).

**Figure 4:**
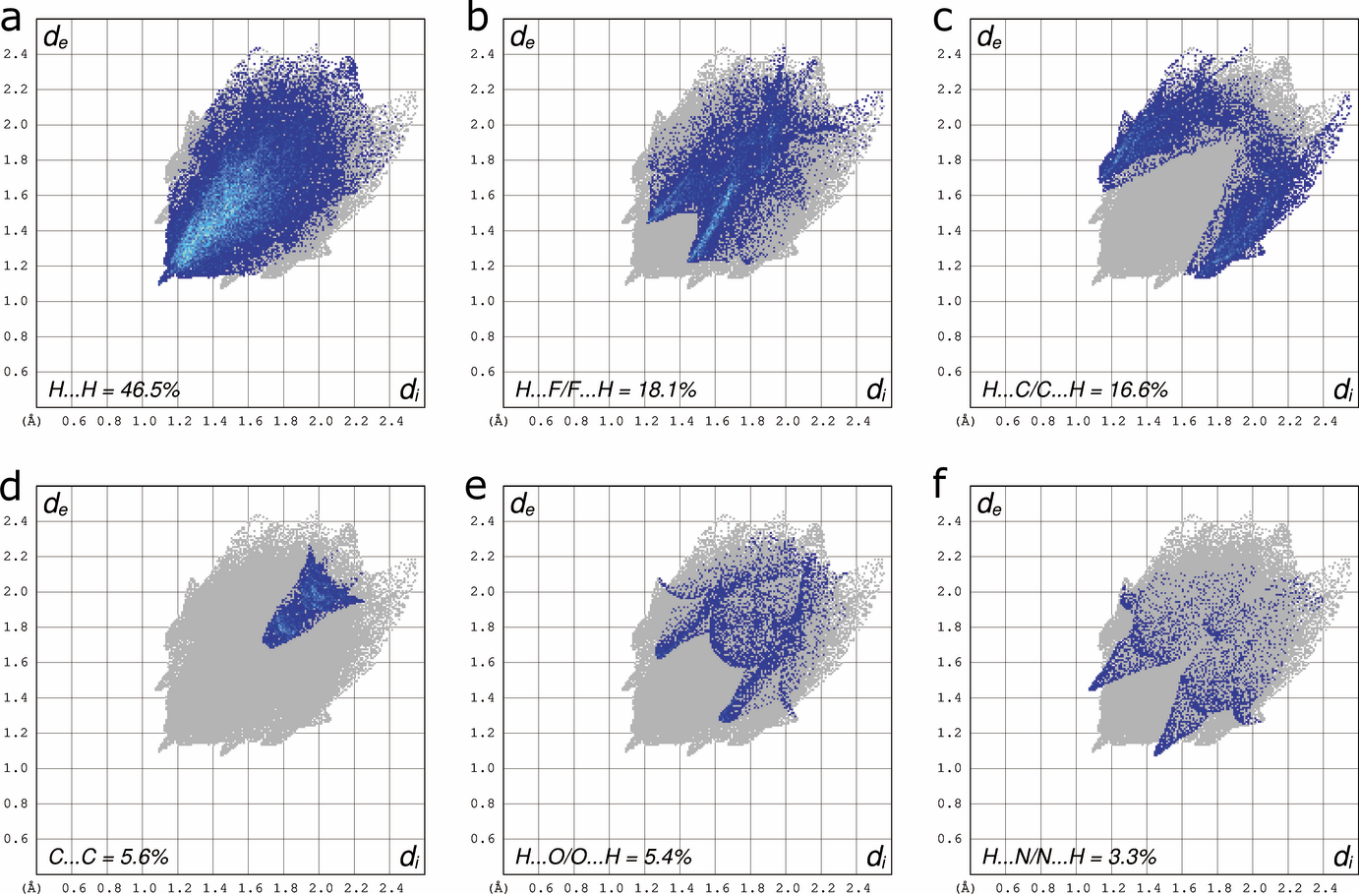
Two-dimensional fingerprint plots qu­anti­fying the various atom–atom contact coverages present in the crystal packing: (*a*) H⋯H = 46.5%; (*b*) H⋯F/F⋯H = 18.1%; (*c*) H⋯C/C⋯H = 16.6%; (*d*) C⋯C = 5.6%; (*e*) H⋯O/O⋯H = 5.4%; (*f*) H⋯N/N⋯H = 3.3%.

There are no especially strong inter­molecular inter­actions in the crystal packing of **R13**. The default geometric search for hydrogen-bond type contacts in *SHELXL* (Sheldrick, 2015*b* ▸) and *Mercury* (Macrae *et al.*, 2020 ▸) suggests no potential inter­molecular hydrogen bonds. A plot of the Hirshfeld surface (HS) mapped over *d*_norm_ (Fig. 2 ▸*a*) in *CrystalExplorer21* (Spackman *et al.*, 2021 ▸), however, reveals a pair of small red spots representing close contacts of the form C19^i^—H19^i^⋯N1 [symmetry code: (i) = 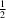 − *x*, 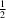 + *y*, 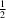 − *z*]. These are necessarily weak, as evident from the *D*⋯*A* distance and *D*—H⋯*A* angle given in Table 1 ▸. The remainder of the HS mapped over *d_norm_* is a largely featureless expanse of blue and white (contact distances larger than and equal to the sum of van der Waals radii, respectively). The HS mapped over ‘shape index’ (Fig. 2 ▸*b*), however, reveals pairs of juxtaposed, roughly triangular, blue and red regions that are a characteristic signature of π–π-stacking inter­actions (Tan *et al.*, 2019 ▸). The inter­planar separation of oxazole ring N1–C1–O1–C2–C3 to its inversion-related counterpart [*via* symmetry operation (ii) 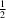 − *x*, 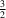 − *y*, 1 − *z*] is 3.3426 (11) Å. Mutual overlap of benzene rings C4–C9 and C16^ii^–C21^ii^ (and *vice versa*) is less distinct; the centroid–centroid distance is 3.9439 (11) Å and the rings are not parallel, but mis-aligned by 11.56 (6)° (Table 1 ▸). The manner in which these π–π inter­actions as well as the weak hydrogen-bond-like contacts combine in the crystal packing is shown in Fig. 3 ▸. Hirshfeld surface fingerprint plots qu­anti­fying the atom–atom contact coverages are given in Fig. 4 ▸, showing that the vast majority of inter­molecular contacts involve hydrogen.

## 4. Database survey

A search of the CSD (v5.45 with updates as of March 2024; Groom *et al.*, 2016 ▸) using a fragment consisting of the three rings of **R13**, but with the fluorine, eth­oxy, and *t*-butyl substituents removed and the double bonds of the oxazole ring specified as ‘any’ type of bond, returned 336 hits. The latter criterion ensures that entries with both oxazole and di­hydro-oxazole five-membered rings would be caught. A pared-down fragment without eth­oxy or *t*-butyl, but with the two fluorine atoms included gave just two matches, CSD refcodes DOGMEV and LIYZUS. Structure DOGMEV (Roque *et al.*, 2023 ▸), or 4-(4-*t*-butyl-2-eth­oxy­phen­yl)-2-[4-(5,5-dimethyl-1,3,2-dioxaborinan-2-yl)-2,6-di­fluoro­phen­yl]-4,5-di­hydro-1,3-oxazole, has a di­methyl­dioxaborinanyl group attached at the 4-position of the difluorinated benzene ring and a di­hydro-oxazole five-membered ring (*i.e.*, just one double bond, as in etoxazole). Structure LIYZUS (Saha *et al.*, 2023 ▸) is 5-eth­oxy-2-(penta­fluoro­phen­yl)-4-phenyl-1,3-oxazole, which has an oxazole ring (*i.e*., two double bonds) as per **R13**, but with a penta­fluoro­phenyl ring at the oxazole 2-position, an unsubstituted phenyl at the 4-position and an eth­oxy group at the 5-position.

## 5. Synthesis and crystallization

5.0 g of etoxazole were placed in a 100 mL round-bottom flask and heated in a controlled manner at 2 K min^−1^ to 377 K, *i.e.*, just past its melting point (374–375 K). After cooling to RT, the resulting solid was dissolved in 10 ml of 100% hexane. The resulting solution containing about 40% etoxazole, 40% of the **R13** metabolite, and 20% unknown products was purified by column chromatography using 100% hexane as the mobile phase and 60-120 mesh size silica gel as the stationary phase. It was then recrystallized from 100% hexane, giving crystals of **R13** suitable for X-ray analysis.

NMR spectra were recorded on an SA-AGILENT 400 MHz NMR spectrometer: ^1^H NMR: CDCl_3_ (400 MHz, δ ppm): 1.310–1.313 [*s*, 9H, C(CH_3_)_3_]; 1.524–1.559 (*t*, 3H, *J* = 6.8 Hz, CH_3_); 4.176–4.228 (*q*, 2H, *J* = 7.2 Hz, CH_2_); 6.96–7.09 (*m*, 4H, aromatic); 7.375–7.417 (*m*, 1H, aromatic); 8.116–8.135 (*d*, 1H, aromatic); 8.326 (*s*, 1H, oxazole).

## 6. Refinement

Crystal data, data collection, and structure refinement details are given in Table 2 ▸. All hydrogens were present in difference-Fourier maps, but were subsequently included in the refinement using riding models, with constrained distances of 0.95 Å (*R*_2_CH), 0.99 Å (*R*_2_CH_2_) and 0.98 Å (*R*CH_3_). *U*_iso_(H) parameters were set to either 1.2*U*_eq_ or 1.5*U*_eq_ (*R*CH_3_ only) of the attached carbon.

## Supplementary Material

Crystal structure: contains datablock(s) I, global. DOI: 10.1107/S2056989024010600/nx2015sup1.cif

Structure factors: contains datablock(s) I. DOI: 10.1107/S2056989024010600/nx2015Isup2.hkl

Supporting information file. DOI: 10.1107/S2056989024010600/nx2015Isup3.cml

CCDC reference: 2397916

Additional supporting information:  crystallographic information; 3D view; checkCIF report

## Figures and Tables

**Table 1:** Close contacts (Å, °) for **R13**

Weak hydrogen bonds				
*D*—H⋯*A*	*D*—H	H⋯*A*	*D*⋯*A*	*D*—H⋯*A*
C2—H2⋯O2	0.95	2.29	2.7807 (12)	111.1
C19^i^—H19^i^⋯N	0.95	2.61	3.3494 (14)	134.7
π–π stacks				
*Ring 1⋯ring 2*		*Distance*	*Dihedral angle*	
Ox⋯Ox^ii^(inter­planar)		3.3426 (11)	0 (parallel)	
*Cg*(Ox)⋯*Cg*(Ox)^ii^		3.3894 (11)	0 (parallel)	
*Cg*(C4–C9)⋯*Cg*(C16–C21)^ii^		3.9439 (11)	11.56 (6)	

Abbreviations: Ox = oxazole; *Cg* = centroid. Symmetry codes: (i) = 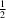 − *x*, 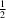 + *y*, 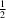 − *z*; (ii) = 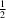 − *x*, 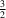 − *y*, 1 − *z*.

**Table 2:** Experimental details

Crystal data	
Chemical formula	C_21_H_21_F_2_NO_2_
*M*_r_	357.39
Crystal system, space group	Monoclinic, *C*2/*c*
Temperature (K)	100
*a*, *b*, *c* (Å)	18.4793 (6), 10.4036 (3), 18.5669 (7)
β (°)	93.035 (1)
*V* (Å^3^)	3564.5 (2)
*Z*	8
Radiation type	Mo *K*α
μ (mm^−1^)	0.10
Crystal size (mm)	0.20 × 0.19 × 0.11

Data collection	
Diffractometer	Bruker D8 Venture dual source
Absorption correction	Multi-scan (*SADABS*; Krause *et al.*, 2015 ▸)
*T*_min_, *T*_max_	0.904, 0.959
No. of measured, independent and observed [*I* > 2σ(*I*)] reflections	41370, 4095, 3558
*R*_int_	0.061
(sin θ/λ)_max_ (Å^−1^)	0.650

Refinement	
*R*[*F*^2^ > 2σ(*F*^2^)], *wR*(*F*^2^), *S*	0.033, 0.085, 1.07
No. of reflections	4095
No. of parameters	239
H-atom treatment	H-atom parameters constrained
Δρ_max_, Δρ_min_ (e Å^−3^)	0.26, −0.22

Computer programs: *APEX5* (Bruker, 2023 ▸), *SHELXT* (Sheldrick, 2015*a* ▸), *SHELXL2019/2* (Sheldrick, 2015*b* ▸), *XP* in *SHELXTL* (Sheldrick, 2008 ▸), *SHELX* (Sheldrick, 2008 ▸) and *publCIF* (Westrip, 2010 ▸).

## supplementary crystallographic information

### 4-(4-tert-Butyl-2-ethoxyphenyl)-2-(2,6-difluorophenyl)oxazole Crystal data

**Table:**

C_21_H_21_F_2_NO_2_	*F*(000) = 1504
*M**_r_* = 357.39	*D*_x_ = 1.332 Mg m^−^^3^
Monoclinic, *C*2/*c*	Mo *K*α radiation, λ = 0.71073 Å
*a* = 18.4793 (6) Å	Cell parameters from 9910 reflections
*b* = 10.4036 (3) Å	θ = 2.3–27.5°
*c* = 18.5669 (7) Å	µ = 0.10 mm^−^^1^
β = 93.035 (1)°	*T* = 100 K
*V* = 3564.5 (2) Å^3^	Cut block, colourless
*Z* = 8	0.20 × 0.19 × 0.11 mm

### 4-(4-tert-Butyl-2-ethoxyphenyl)-2-(2,6-difluorophenyl)oxazole Data collection

**Table:**

Bruker D8 Venture dual source diffractometer	4095 independent reflections
Radiation source: microsource	3558 reflections with *I* > 2σ(*I*)
Detector resolution: 7.41 pixels mm^-1^	*R*_int_ = 0.061
φ and ω scans	θ_max_ = 27.5°, θ_min_ = 2.2°
Absorption correction: multi-scan (*SADABS*; Krause *et al*., 2015)	*h* = −23→23
*T*_min_ = 0.904, *T*_max_ = 0.959	*k* = −13→13
41370 measured reflections	*l* = −24→24

### 4-(4-tert-Butyl-2-ethoxyphenyl)-2-(2,6-difluorophenyl)oxazole Refinement

**Table:**

Refinement on *F*^2^	Primary atom site location: structure-invariant direct methods
Least-squares matrix: full	Secondary atom site location: difference Fourier map
*R*[*F*^2^ > 2σ(*F*^2^)] = 0.033	Hydrogen site location: difference Fourier map
*wR*(*F*^2^) = 0.085	H-atom parameters constrained
*S* = 1.07	*w* = 1/[σ^2^(*F*_o_^2^) + (0.0317*P*)^2^ + 1.7737*P*] where *P* = (*F*_o_^2^ + 2*F*_c_^2^)/3
4095 reflections	(Δ/σ)_max_ < 0.001
239 parameters	Δρ_max_ = 0.26 e Å^−^^3^
0 restraints	Δρ_min_ = −0.22 e Å^−^^3^

### 4-(4-tert-Butyl-2-ethoxyphenyl)-2-(2,6-difluorophenyl)oxazole Special details

**Table:**

Experimental. The crystal was mounted using polyisobutene oil on the tip of a fine glass fibre, which was fastened in a copper mounting pin with electrical solder. It was placed directly into the cold gas stream of a liquid-nitrogen based cryostat (Hope, 1994; Parkin & Hope, 1998). Diffraction data were collected with the crystal at 100K.
Geometry. All esds (except the esd in the dihedral angle between two l.s. planes) are estimated using the full covariance matrix. The cell esds are taken into account individually in the estimation of esds in distances, angles and torsion angles; correlations between esds in cell parameters are only used when they are defined by crystal symmetry. An approximate (isotropic) treatment of cell esds is used for estimating esds involving l.s. planes.
Refinement. Refinement progress was checked using *Platon* (Spek, 2020) and by an *R*-tensor (Parkin, 2000). The final model was further checked with the IUCr utility *checkCIF*.

### 4-(4-tert-Butyl-2-ethoxyphenyl)-2-(2,6-difluorophenyl)oxazole Fractional atomic coordinates and isotropic or equivalent isotropic displacement parameters (Å^2^)

**Table:**

	*x*	*y*	*z*	*U*_iso_*/*U*_eq_	
F1	0.31274 (3)	0.82135 (6)	0.32509 (3)	0.02734 (16)	
F2	0.05890 (3)	0.81737 (7)	0.34205 (4)	0.03608 (19)	
O1	0.14316 (4)	0.71474 (7)	0.44635 (4)	0.02199 (17)	
O2	0.19533 (4)	0.41164 (7)	0.58364 (4)	0.02220 (17)	
N1	0.24848 (5)	0.64149 (8)	0.41010 (5)	0.02001 (18)	
C1	0.19516 (5)	0.72003 (9)	0.39718 (5)	0.0190 (2)	
C2	0.16811 (6)	0.62314 (10)	0.49472 (6)	0.0216 (2)	
H2	0.144270	0.596124	0.536213	0.026*	
C3	0.23218 (5)	0.57765 (9)	0.47364 (5)	0.0189 (2)	
C4	0.28343 (5)	0.48460 (9)	0.50675 (5)	0.0190 (2)	
C5	0.35428 (6)	0.47979 (10)	0.48489 (6)	0.0216 (2)	
H5	0.368284	0.535977	0.447771	0.026*	
C6	0.40484 (6)	0.39551 (10)	0.51568 (6)	0.0229 (2)	
H6	0.452820	0.395248	0.499675	0.028*	
C7	0.38619 (5)	0.31068 (9)	0.57009 (5)	0.0196 (2)	
C8	0.31520 (5)	0.31386 (9)	0.59192 (5)	0.0197 (2)	
H8	0.301032	0.255850	0.628094	0.024*	
C9	0.26455 (5)	0.40030 (9)	0.56181 (5)	0.0190 (2)	
C10	0.17146 (6)	0.3233 (1)	0.63687 (6)	0.0222 (2)	
H10A	0.205099	0.324699	0.680171	0.027*	
H10B	0.169803	0.234787	0.617268	0.027*	
C11	0.09681 (6)	0.36551 (12)	0.65605 (6)	0.0294 (3)	
H11A	0.063603	0.360211	0.613215	0.044*	
H11B	0.098855	0.454391	0.673527	0.044*	
H11C	0.079477	0.309378	0.693857	0.044*	
C12	0.44366 (5)	0.22003 (10)	0.60412 (6)	0.0216 (2)	
C13	0.41297 (6)	0.12946 (11)	0.65984 (6)	0.0283 (2)	
H13A	0.373991	0.077640	0.636764	0.043*	
H13B	0.393756	0.180052	0.699046	0.043*	
H13C	0.451514	0.072699	0.679385	0.043*	
C14	0.50460 (6)	0.30006 (12)	0.64139 (7)	0.0315 (3)	
H14A	0.525169	0.357895	0.606173	0.047*	
H14B	0.542537	0.242579	0.661459	0.047*	
H14C	0.484974	0.350789	0.680327	0.047*	
C15	0.47507 (6)	0.13723 (11)	0.54504 (6)	0.0282 (2)	
H15A	0.435806	0.090190	0.519134	0.042*	
H15B	0.509964	0.075983	0.567013	0.042*	
H15C	0.499521	0.192584	0.511211	0.042*	
C16	0.18637 (5)	0.81374 (9)	0.33852 (5)	0.0192 (2)	
C17	0.24577 (5)	0.86239 (10)	0.30370 (5)	0.0203 (2)	
C18	0.23993 (6)	0.95121 (10)	0.24873 (6)	0.0227 (2)	
H18	0.281992	0.981741	0.226916	0.027*	
C19	0.17194 (6)	0.99543 (10)	0.22567 (6)	0.0241 (2)	
H19	0.167056	1.057008	0.187945	0.029*	
C20	0.11108 (6)	0.94965 (11)	0.25776 (6)	0.0264 (2)	
H20	0.064166	0.978656	0.241898	0.032*	
C21	0.11937 (6)	0.86175 (10)	0.31282 (6)	0.0237 (2)	

### 4-(4-tert-Butyl-2-ethoxyphenyl)-2-(2,6-difluorophenyl)oxazole Atomic displacement parameters (Å^2^)

**Table:**

	*U*^11^	*U*^22^	*U*^33^	*U*^12^	*U*^13^	*U*^23^
F1	0.0184 (3)	0.0327 (3)	0.0312 (3)	0.0040 (3)	0.0041 (2)	0.0080 (3)
F2	0.0179 (3)	0.0475 (4)	0.0429 (4)	−0.0016 (3)	0.0019 (3)	0.0198 (3)
O1	0.0204 (4)	0.0240 (4)	0.0219 (4)	0.0021 (3)	0.0036 (3)	0.0044 (3)
O2	0.0174 (3)	0.0247 (4)	0.0250 (4)	0.0020 (3)	0.0051 (3)	0.0068 (3)
N1	0.0217 (4)	0.0190 (4)	0.0194 (4)	0.0001 (3)	0.0017 (3)	0.0007 (3)
C1	0.0186 (5)	0.0191 (5)	0.0193 (5)	−0.0016 (4)	0.0020 (4)	−0.0013 (4)
C2	0.0225 (5)	0.0223 (5)	0.0199 (5)	0.0006 (4)	0.0020 (4)	0.0042 (4)
C3	0.0208 (5)	0.0178 (4)	0.0181 (5)	−0.0025 (4)	0.0009 (4)	−0.0005 (4)
C4	0.0202 (5)	0.0176 (4)	0.0191 (5)	0.0000 (4)	0.0008 (4)	−0.0013 (4)
C5	0.0235 (5)	0.0208 (5)	0.0209 (5)	−0.0008 (4)	0.0040 (4)	0.0020 (4)
C6	0.0184 (5)	0.0252 (5)	0.0254 (5)	0.0005 (4)	0.0036 (4)	0.0005 (4)
C7	0.0197 (5)	0.0195 (5)	0.0195 (5)	0.0009 (4)	0.0004 (4)	−0.0028 (4)
C8	0.0211 (5)	0.0190 (5)	0.0190 (5)	−0.0004 (4)	0.0018 (4)	0.0001 (4)
C9	0.0178 (5)	0.0201 (5)	0.0193 (5)	−0.0010 (4)	0.0019 (4)	−0.0019 (4)
C10	0.0217 (5)	0.0223 (5)	0.0227 (5)	−0.0011 (4)	0.0026 (4)	0.0056 (4)
C11	0.0224 (5)	0.0364 (6)	0.0297 (6)	0.0012 (5)	0.0049 (4)	0.0118 (5)
C12	0.0193 (5)	0.0239 (5)	0.0216 (5)	0.0028 (4)	0.0008 (4)	0.0000 (4)
C13	0.0254 (5)	0.0316 (6)	0.0282 (6)	0.0072 (5)	0.0031 (4)	0.0078 (5)
C14	0.0239 (6)	0.0360 (6)	0.0337 (6)	0.0001 (5)	−0.0053 (5)	−0.0009 (5)
C15	0.0284 (6)	0.0287 (6)	0.0277 (6)	0.0084 (5)	0.0048 (4)	0.0001 (4)
C16	0.0218 (5)	0.0180 (5)	0.0179 (5)	0.0001 (4)	0.0008 (4)	−0.0011 (4)
C17	0.0188 (5)	0.0212 (5)	0.0210 (5)	0.0023 (4)	0.0014 (4)	−0.0025 (4)
C18	0.0249 (5)	0.0224 (5)	0.0212 (5)	−0.0011 (4)	0.0055 (4)	−0.0005 (4)
C19	0.0305 (6)	0.0210 (5)	0.0209 (5)	0.0011 (4)	0.0010 (4)	0.0021 (4)
C20	0.0233 (5)	0.0281 (5)	0.0276 (6)	0.0037 (4)	−0.0017 (4)	0.0041 (4)
C21	0.0195 (5)	0.0255 (5)	0.0265 (5)	−0.0020 (4)	0.0032 (4)	0.0023 (4)

### 4-(4-tert-Butyl-2-ethoxyphenyl)-2-(2,6-difluorophenyl)oxazole Geometric parameters (Å, º)

**Table:**

F1—C17	1.3489 (12)	C11—H11A	0.9800
F2—C21	1.3495 (12)	C11—H11B	0.9800
O1—C1	1.3611 (12)	C11—H11C	0.9800
O1—C2	1.3725 (12)	C12—C13	1.5303 (15)
O2—C9	1.3671 (12)	C12—C15	1.5328 (15)
O2—C10	1.4361 (12)	C12—C14	1.5355 (15)
N1—C1	1.2927 (13)	C13—H13A	0.9800
N1—C3	1.4004 (13)	C13—H13B	0.9800
C1—C16	1.4644 (14)	C13—H13C	0.9800
C2—C3	1.3521 (14)	C14—H14A	0.9800
C2—H2	0.9500	C14—H14B	0.9800
C3—C4	1.4664 (14)	C14—H14C	0.9800
C4—C5	1.3919 (14)	C15—H15A	0.9800
C4—C9	1.4049 (14)	C15—H15B	0.9800
C5—C6	1.3827 (15)	C15—H15C	0.9800
C5—H5	0.9500	C16—C21	1.3955 (15)
C6—C7	1.3983 (14)	C16—C17	1.3978 (14)
C6—H6	0.9500	C17—C18	1.3768 (14)
C7—C8	1.3938 (14)	C18—C19	1.3843 (15)
C7—C12	1.5314 (14)	C18—H18	0.9500
C8—C9	1.3934 (14)	C19—C20	1.3848 (16)
C8—H8	0.9500	C19—H19	0.9500
C10—C11	1.5081 (15)	C20—C21	1.3741 (15)
C10—H10A	0.9900	C20—H20	0.9500
C10—H10B	0.9900		
			
C1—O1—C2	104.02 (8)	C13—C12—C7	112.60 (8)
C9—O2—C10	118.37 (8)	C13—C12—C15	107.80 (9)
C1—N1—C3	105.08 (8)	C7—C12—C15	109.36 (8)
N1—C1—O1	114.10 (9)	C13—C12—C14	108.56 (9)
N1—C1—C16	127.75 (9)	C7—C12—C14	109.15 (9)
O1—C1—C16	118.12 (9)	C15—C12—C14	109.32 (9)
C3—C2—O1	108.82 (9)	C12—C13—H13A	109.5
C3—C2—H2	125.6	C12—C13—H13B	109.5
O1—C2—H2	125.6	H13A—C13—H13B	109.5
C2—C3—N1	107.97 (9)	C12—C13—H13C	109.5
C2—C3—C4	131.74 (9)	H13A—C13—H13C	109.5
N1—C3—C4	120.22 (9)	H13B—C13—H13C	109.5
C5—C4—C9	117.61 (9)	C12—C14—H14A	109.5
C5—C4—C3	119.87 (9)	C12—C14—H14B	109.5
C9—C4—C3	122.51 (9)	H14A—C14—H14B	109.5
C6—C5—C4	121.84 (10)	C12—C14—H14C	109.5
C6—C5—H5	119.1	H14A—C14—H14C	109.5
C4—C5—H5	119.1	H14B—C14—H14C	109.5
C5—C6—C7	120.8 (1)	C12—C15—H15A	109.5
C5—C6—H6	119.6	C12—C15—H15B	109.5
C7—C6—H6	119.6	H15A—C15—H15B	109.5
C8—C7—C6	117.83 (9)	C12—C15—H15C	109.5
C8—C7—C12	122.52 (9)	H15A—C15—H15C	109.5
C6—C7—C12	119.65 (9)	H15B—C15—H15C	109.5
C9—C8—C7	121.41 (9)	C21—C16—C17	114.61 (9)
C9—C8—H8	119.3	C21—C16—C1	123.64 (9)
C7—C8—H8	119.3	C17—C16—C1	121.75 (9)
O2—C9—C8	123.77 (9)	F1—C17—C18	117.73 (9)
O2—C9—C4	115.73 (9)	F1—C17—C16	118.68 (9)
C8—C9—C4	120.49 (9)	C18—C17—C16	123.59 (10)
O2—C10—C11	107.11 (8)	C17—C18—C19	119.12 (10)
O2—C10—H10A	110.3	C17—C18—H18	120.4
C11—C10—H10A	110.3	C19—C18—H18	120.4
O2—C10—H10B	110.3	C18—C19—C20	119.8 (1)
C11—C10—H10B	110.3	C18—C19—H19	120.1
H10A—C10—H10B	108.5	C20—C19—H19	120.1
C10—C11—H11A	109.5	C21—C20—C19	119.2 (1)
C10—C11—H11B	109.5	C21—C20—H20	120.4
H11A—C11—H11B	109.5	C19—C20—H20	120.4
C10—C11—H11C	109.5	F2—C21—C20	117.70 (9)
H11A—C11—H11C	109.5	F2—C21—C16	118.60 (9)
H11B—C11—H11C	109.5	C20—C21—C16	123.68 (10)
			
C3—N1—C1—O1	−0.50 (11)	C3—C4—C9—C8	−179.69 (9)
C3—N1—C1—C16	177.39 (9)	C9—O2—C10—C11	174.49 (9)
C2—O1—C1—N1	0.39 (11)	C8—C7—C12—C13	3.86 (14)
C2—O1—C1—C16	−177.71 (9)	C6—C7—C12—C13	−177.08 (9)
C1—O1—C2—C3	−0.11 (11)	C8—C7—C12—C15	123.67 (10)
O1—C2—C3—N1	−0.18 (11)	C6—C7—C12—C15	−57.26 (12)
O1—C2—C3—C4	176.69 (10)	C8—C7—C12—C14	−116.78 (11)
C1—N1—C3—C2	0.40 (11)	C6—C7—C12—C14	62.29 (12)
C1—N1—C3—C4	−176.89 (9)	N1—C1—C16—C21	156.63 (11)
C2—C3—C4—C5	−162.47 (11)	O1—C1—C16—C21	−25.56 (14)
N1—C3—C4—C5	14.08 (14)	N1—C1—C16—C17	−23.42 (16)
C2—C3—C4—C9	15.99 (17)	O1—C1—C16—C17	154.39 (9)
N1—C3—C4—C9	−167.46 (9)	C21—C16—C17—F1	179.95 (9)
C9—C4—C5—C6	0.07 (15)	C1—C16—C17—F1	−0.01 (14)
C3—C4—C5—C6	178.60 (9)	C21—C16—C17—C18	0.45 (15)
C4—C5—C6—C7	0.45 (16)	C1—C16—C17—C18	−179.51 (9)
C5—C6—C7—C8	0.16 (15)	F1—C17—C18—C19	−179.85 (9)
C5—C6—C7—C12	−178.95 (9)	C16—C17—C18—C19	−0.35 (16)
C6—C7—C8—C9	−1.30 (15)	C17—C18—C19—C20	−0.28 (16)
C12—C7—C8—C9	177.78 (9)	C18—C19—C20—C21	0.77 (16)
C10—O2—C9—C8	−4.80 (14)	C19—C20—C21—F2	−178.85 (10)
C10—O2—C9—C4	176.53 (9)	C19—C20—C21—C16	−0.67 (17)
C7—C8—C9—O2	−176.75 (9)	C17—C16—C21—F2	178.24 (9)
C7—C8—C9—C4	1.86 (15)	C1—C16—C21—F2	−1.81 (16)
C5—C4—C9—O2	177.51 (9)	C17—C16—C21—C20	0.07 (16)
C3—C4—C9—O2	−0.98 (14)	C1—C16—C21—C20	−179.98 (10)
C5—C4—C9—C8	−1.20 (14)		

### 4-(4-tert-Butyl-2-ethoxyphenyl)-2-(2,6-difluorophenyl)oxazole Hydrogen-bond geometry (Å, º)

**Table:**

*D*—H···*A*	*D*—H	H···*A*	*D*···*A*	*D*—H···*A*
C2—H2···O2	0.95	2.29	2.7807 (12)	111
